# Chondroitin Sulfate/Dermatan Sulfate Hybrid Chains from Swim Bladder: Isolation, Structural Analysis, and Anticoagulant Activity

**DOI:** 10.3390/md22010009

**Published:** 2023-12-21

**Authors:** Yue Yao, Hao Tang, Haiqiong Ma, Zidong Liu, Jinwen Huang, Xiufen Yang, Longyan Zhao, Qingxia Yuan

**Affiliations:** 1 Guangxi Key Laboratory of Marine Drugs, Institute of Marine Drugs, Guangxi University of Chinese Medicine, Nanning 530200, China; kuchenivo@foxmail.com (Y.Y.); yaoxuetanghao@outlook.com (H.T.); mhq18878839254@163.com (H.M.); lzdcodeero@126.com (Z.L.); huangjinwen1127@163.com (J.H.); 2 School of Pharmacy, Guangxi University of Chinese Medicine, Nanning 530200, China

**Keywords:** swim bladder, glycosaminoglycan, CS/DS hybrid chain, structure, anticoagulant activity

## Abstract

Glycosaminoglycans (GAGs) with unique structures from marine animals show intriguing pharmacological activities and negligible biological risks, providing more options for us to explore safer agents. The swim bladder is a tonic food and folk medicine, and its GAGs show good anticoagulant activity. In this study, two GAGs, CMG-1.0 and GMG-1.0, were extracted and isolated from the swim bladder of *Cynoscion microlepidotus* and *Gadus morhua*. The physicochemical properties, precise structural characteristics, and anticoagulant activities of these GAGs were determined for the first time. The analysis results of the CMG-1.0 and GMG-1.0 showed that they were chondroitin sulfate (CS)/dermatan sulfate (DS) hybrid chains with molecular weights of 109.3 kDa and 123.1 kDa, respectively. They were mainly composed of the repeating disaccharide unit of -{IdoA-α1,3-GalNAc_4S_-β1,4-}- (DS-A). The DS-B disaccharide unit of -{IdoA_2S_-α1,3-GalNAc_4S_-β1,4-}- also existed in both CMG-1.0 and GMG-1.0. CMG-1.0 had a higher proportion of CS-O disaccharide unit -{-GlcA-β1,3-GalNAc-β1,4-}- but a lower proportion of CS-E disaccharide unit -{-GlcA-β1,3-GalNAc_4S6S_-β1,4-}- than GMG-1.0. The disaccharide compositions of the GAGs varied in a species-specific manner. Anticoagulant activity assay revealed that both CMG-1.0 and GMG-1.0 had potent anticoagulant activity, which can significantly prolong activated partial thromboplastin time. GMG-1.0 also can prolong the thrombin time. CMG-1.0 showed no intrinsic tenase inhibition activity, while GMG-1.0 can obviously inhibit intrinsic tenase with EC_50_ of 58 nM. Their significantly different anticoagulant activities may be due to their different disaccharide structural units and proportions. These findings suggested that swim bladder by-products of fish processing of these two marine organisms may be used as a source of anticoagulants.

## 1. Introduction

Glycosaminoglycans (GAGs) are complex acidic polysaccharides composed of repeating disaccharide units formed by hexosamine and uronic acid (or galactose (Gal)). The common GAGs can be divided into chondroitin sulfate (CS), dermatan sulfate (DS), heparin (HP), heparan sulfate (HS), hyaluronic acid (HA), and keratan sulfate (KS) according to their monosaccharide compositions and sulfate substitution positions. GAGs are widely distributed in the animal kingdom, and their structures are related to animal tissues, organs, and species [1,2]. The structural complexity of GAGs results in their diverse activities. Various GAGs have been isolated and found to possess anticoagulant, antithrombotic, nerve regeneration, and anti-inflammatory activities [3]. The most typical example is heparin, a very famous GAG, which has been widely used as an anticoagulant in clinical for over eighty years [4,5]. Another widely known GAG is CS, which is used in the treatment of joint diseases [6]. HA has also been used in skin regeneration, wound healing, and cosmetic fields [7]. The wide application of these GAGs encourages researchers to look for GAGs from different animal sources with unique structures and remarkable activity.

Numerous commercially available GAGs, including HP, are extracted from terrestrial mammalian tissues, such as bovine lung and porcine intestine. However, they have some inevitable problems, including religious concerns and the potential risk of contamination by pathogens, such as prion virus and African swine fever virus [8]. Marine animal resources are abundant, and most marine animals contain GAGs with novel structures, intriguing pharmacological functions, and negligible biological risks, which provide more options for us to explore safer agents [9,10]. For example, a low-molecular-weight fucosylated CS with weight mean molecular mass (*M*_w_) of 5300 Da (named LFG-53) derived from sea cucumber has been prepared as a novel anticoagulant with low side effects and approved by the FDA for clinical study [10]. However, preparing this anticoagulant depends on the high cost of raw materials (sea cucumbers). Therefore, searching for novel GAG compounds from other marine organisms with potent anticoagulant activity is still a focus of research for the development of new anticoagulants.

The swim bladder is one of the by-products of fish processing and has long been used as not only tonic food but also folk medicine in Asia, particularly in southern China [11]. The swim bladder weight accounts for ~1.3% of the final fish weight [12], and the scale of swim bladder production in Asia is quite large. The swim bladders from some fishes are dried and sold as fish maw, which has a great market demand for their high nutritional values and good pharmacological activities [13]. Collagen and peptides are the major components of the swim bladder and have been studied extensively [14,15,16]. Some polysaccharides have also been isolated from the swim bladder and found to possess preventive effects on gastric injury, therapeutic effects on lupus nephritis, and anticancer activity [17,18,19]. However, the structural information of these polysaccharides is very limited. In recent years, GAGs from swim bladders were isolated, and their basic structural characteristics were analyzed. For example, in 2018, the disaccharide compositions of swim bladder GAG were analyzed by compositional analysis of GAG disaccharides using heparin lyase I, II, III, and chondroitin lyase ABC and by ^1^H NMR spectroscopy, indicating that the GAG from the commercial dried fish maw, “hudiejiao”, consisted of CS (95%) and HS (5%) of the total GAG [20]. Subsequently, GAGs isolated from the swim bladders of *Lateolabrax japonicus* and *Aristichthys nobili* were considered to be CS-A mainly constituted by the repeating disaccharide unit -{GlcA-β1,3-GalNAc_4S_-β1,4-}-, where GlcA and GalNAc are D-glucuronic acid (GlcA) and N-acetyl-D-galactosamine (GalNAc), respectively [21,22]. However, the precise structures of these GAGs remain to be elucidated. Additionally, these GAGs have exhibited a wide range of activities, such as wound healing, anticoagulant, and anti-inflammatory activities and intervention effects against arsenic-induced damage [20,22,23].

Although several studies have shown that the swim bladder is rich in GAGs, the detailed structure and anticoagulant activity of GAGs from various fish species have not been deeply studied. In this study, the GAG fractions were isolated from the swim bladder of *Cynoscion microlepidotus* (CMG-1.0) and *Gadus morhua* (GMG-1.0) and identified as CS/DS hybrid chains. Further analysis of structure and activity revealed that CMG-1.0 and GMG-1.0 are mainly composed of DS rather than CS units and exhibit potent anticoagulant activity. These findings suggest that CMG-1.0 and GMG-1.0 have potential in the development of anticoagulants, which will facilitate the high-value utilization of the swim bladder resources from fish processing.

## 2. Results and Discussion

### 2.1. Extraction and Purification of CMG-1.0 and GMG-1.0

The yields of crude GAGs extracted from *C. microlepidotus* and *G. morhua* were 0.24% and 0.36% by dry weight of swim bladders, respectively. The crude GAG was further purified by strong anion-exchange chromatography and resulted in two main fractions, CMG-1.0 and GMG-1.0, with yields of 27.4% and 27.9%, respectively, by dry weight of the crude GAGs.

### 2.2. Chemical Compositions of CMG-1.0 and GMG-1.0

The protein content in CMG-1.0 was 0.91 ± 0.55%, and no protein was detected in GMG-1.0 by the Bradford method, indicating that proteins were almost removed by deproteinization and purification. These results were further confirmed by the ultraviolet-visible absorption spectra of CMG-1.0 and GMG-1.0, which showed very weak or no absorption peaks at the wavelength of 280 nm, respectively (Figure S1). The uronic acid contents in CMG-1.0 and GMG-1.0 were 35.04 ± 1.05% and 29.49 ± 1.32%, respectively, and their sulfate contents were 23.04 ± 0.94% and 20.49 ± 0.01%, respectively. The molar ratio of -OSO_3_^−^/-COO^−^ of CMG-1.0 and GMG-1.0 were determined to be 1.16 and 1.56, respectively, by a conductimetric method, which is consistent with the information that CMG-1.0 had a higher content of uronic acid.

### 2.3. Purity and Molecular Weight of CMG-1.0 and GMG-1.0

**Figure 1:**
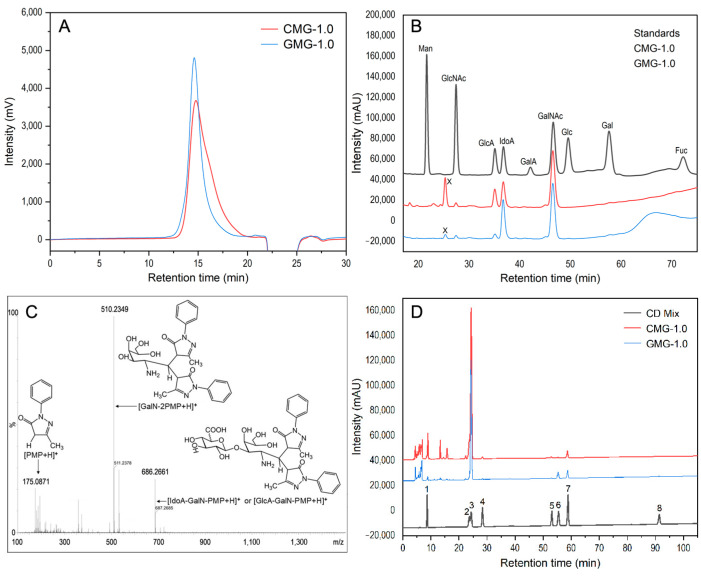
HPGPC profiles of CMG-1.0 and GMG-1.0 (**A**); chromatograms of PMP derivatives of mixed monosaccharide standards, CMG-1.0 and GMG-1.0 (**B**); positive-ion ESI-TOF-MS spectrum (**C**) of PMP-labeled acidolysis-resistant disaccharide (labeled x in **B**); HPLC profiles of disaccharide composition analysis of CMG-1.0 and GMG-1.0 (**D**). The disaccharide standards (**D**) include 1. ΔDi0S (ΔUA-1,3-GalNAc), 2. ΔDi6S (ΔUA-1,3-GalNAc_6S_), 3. ΔDi4S (ΔUA-1,3-GalNAc_4S_), 4. ΔDi2S (ΔUA_2S_-1,3-GalNAc), 5. ΔDi2,6diS (ΔUA_2S_-1,3-GalNAc_6S_), 6. ΔDi4,6diS (ΔUA-1,3-GalNAc_4S6S_), 7. ΔDi2,4diS (ΔUA_2S_-1,3-GalNAc_4S_), and 8. ΔDi2,4,6triS (ΔUA_2S_-1,3-GalNAc_4S6S_).

The purity and *M*_w_ of the samples were determined by high-performance gel permeation chromatography (HPGPC), and the results are shown in Figure 1. Both CMG-1.0 and GMG-1.0 showed a single symmetrical peak, which indicated that they were homogeneous polysaccharides with purity >99% by the area normalization method. The results of cellulose acetate electrophoresis further confirmed the high purity of CMG-1.0 and GMG-1.0 because of only one band shown by these GAGs in the cellulose acetate strip ((Figure S2)). The *M*_w_s of CMG-1.0 and GMG-1.0 were calculated to be 109.3 kDa and 123.1 kDa, respectively, according to their standard curve. Their *M*_w_s were similar to the purified GAG from *Aristichthys nobilis* swim bladder [22] but smaller than the major GAG fraction from a swim bladder, whose species was not identified [20].

### 2.4. Monosaccharide Compositions of CMG-1.0 and GMG-1.0

Monosaccharide compositions of CMG-1.0 and GMG-1.0 are shown in Figure 1B. Both CMG-1.0 and GMG-1.0 were composed of L-iduronic acid (IdoA), GalNAc, GlcA, and N-acetyl-D-glucosamine (GlcNAc) with different molar ratios. The molar ratio of IdoA, GalNAc, GlcA, and GlcNAc was 19.69:22.76:12.48:1.00 for CMG-1.0 and 36.43:26.28:3.95:1.00 for GMG-1.0. In addition, we also observed that there was an unknown peak (labeled x) that appeared at approximately 25 min in the HPLC profiles, which did not match any standard monosaccharide. It was reported that there were acidolysis-resistant disaccharides when GAGs were not sufficiently hydrolyzed [24,25]. The unknown peak may be the disaccharide units derived from CMG-1.0 or GMG-1.0. To prove our hypothesis, the 3-methyl-1-phenyl-2-pyrazolin-5-one (PMP)-derivatized sample was further analyzed by the UPLC-MS, resulting in three pseudo-molecular ions from the unknown peak with mass-to-charge values of 175.0871, 510.2349, and 686.2661, which were consistent with [PMP + H]^+^, [GalNAc-2PMP + H]^+^, and [GlcA/IdoA-GalNAc-PMP + H]^+^, respectively (Figure 1C). Hence, component x was a PMP-labeled disaccharide with a molecular mass of 685.2661 Da, and the main product ion at m/z 510.2349 was due to the glycosidic bond cleavage of GlcA/IdoA-GalNAc-2PMP. The IdoA and GlcA in CMG-1.0 or GMG-1.0 cannot be differentiated by monosaccharide analysis but can be identified by the 1D/2D NMR spectroscopy. Based on the results of monosaccharide composition and cellulose acetate electrophoresis, it can be speculated that both CMG-1.0. and GMG-1.0 may be CS/DS hybrid chains.

### 2.5. Disaccharide Compositions of CMG-1.0 and GMG-1.0

Disaccharide compositions of CMG-1.0 and GMG-1.0 were identified by treatment with chondroitin ABC lyase and analysis of the released unsaturated disaccharides. As shown in Figure 1D, the disaccharide compositions with molar percentages of CMG-1.0 was ΔDi4S (78.61%), ΔDi6S (8.96%), ΔDi0S (7.56%), ΔDi2,4S (3.21%), ΔDi2S (0.78%), ΔDi2,6S (0.64%), and ΔDi4,6S (0.24%). GMG-1.0 also mainly contained the disaccharide ΔDi4S (87.25%) and a small amount of ΔDi4,6S (5.18%), ΔDi2,4S (5.05%), ΔDi0S (1.54%), ΔDi2S (0.37%), ΔDi2,4,6S (0.37%), and ΔDi2,6S (0.24%). Taken together, these data indicated that both CMG-1.0 and GMG-1.0 were mainly composed of ΔDi4S unit, a DS-A disaccharide unit, confirmed by the following NMR analysis. CMG-1.0 had a higher proportion of ΔDi0S units than GMG-1.0, while GMG-1.0 contained a higher proportion of ΔDi4,6S disaccharide units than CMG-1.0. In a previous study, a purified GAG from a commercial swim bladder was determined to have 59.7% of ΔDi4S (CS-A/DS-A) but contain more ΔDi4,6S (36.5%) than CMG-1.0 and GMG-1.0 [20]. Another study reported that a heparin-like GAG from *Aristichthys nobilis* swim bladder was composed almost exclusively of ΔDi4S (CS-A) [22]. Therefore, the disaccharide compositions of GAGs from swim bladders varied in a species-specific manner.

### 2.6. IR Spectrum Analysis of CMG-1.0 and GMG-1.0

The FT-IR spectra of CMG-1.0 and GMG-1.0 are shown in Figure S3. The broad, intense characteristic peaks around 3421 cm^−1^ in CMG-1.0 and 3435 cm^−1^ in GMG-1.0 were due to O-H stretching vibration. The bands around 2926/2943 cm^−1^ were attributed to C-H stretching vibration [26]. The bands around 1630 cm^−1^ and 1418 cm^−1^ were attributed to the stretching vibration of C=O and C-O, respectively, suggesting the existence of uronic acid. Absorptions at approximately 1260 cm^−1^ and 820–860 cm^−1^ were derived from the S=O asymmetric stretching vibration and C-O-S stretching vibration, respectively, indicating the presence of sulfate groups in both GAGs [27]. In addition, the absorption peaks of C-O-S stretching vibration at around 855 cm^−1^ in CMG-1.0 and 843 cm^−1^ in GMG-1.0 indicated that the C-4 position of GalNAc or GlcNAc residues was sulfated [28].

### 2.7. Structural Analysis of CMG-1.0 and GMG-1.0 by NMR Spectroscopy

**Figure 2:**
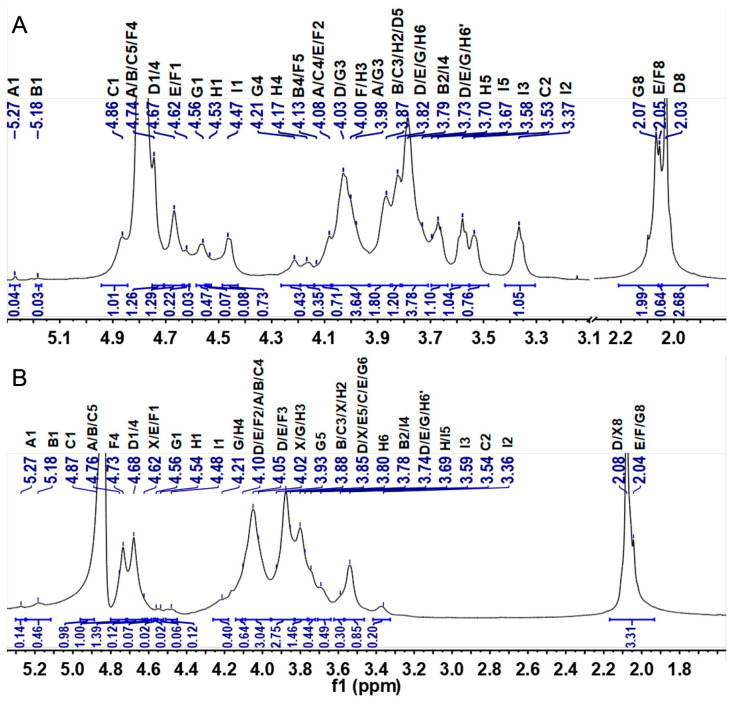
^1^H NMR spectra of CMG-1.0 (**A**) and GMG-1.0 (**B**).

**Figure 3:**
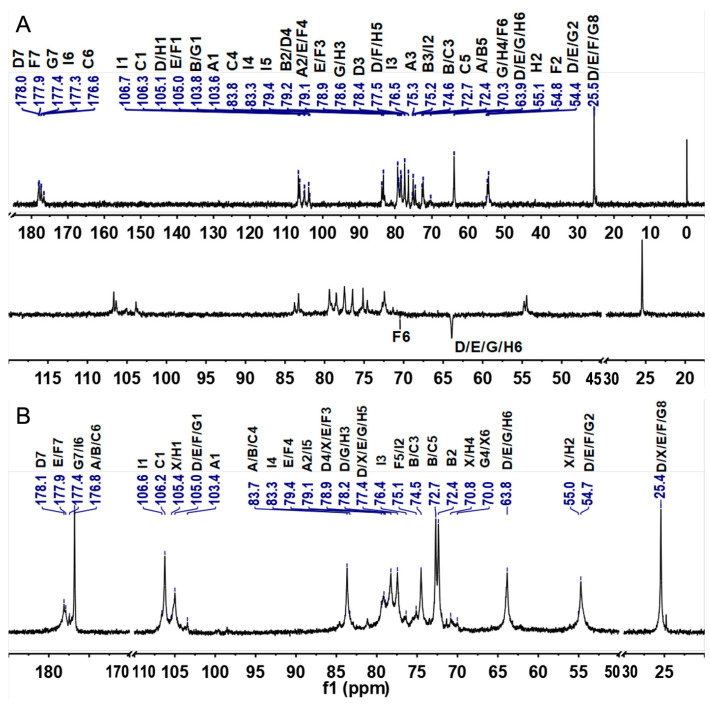
^13^C and DEPT-135 NMR spectra of CMG-1.0 (**A**) and GMG-1.0 (**B**).

**Figure 4:**
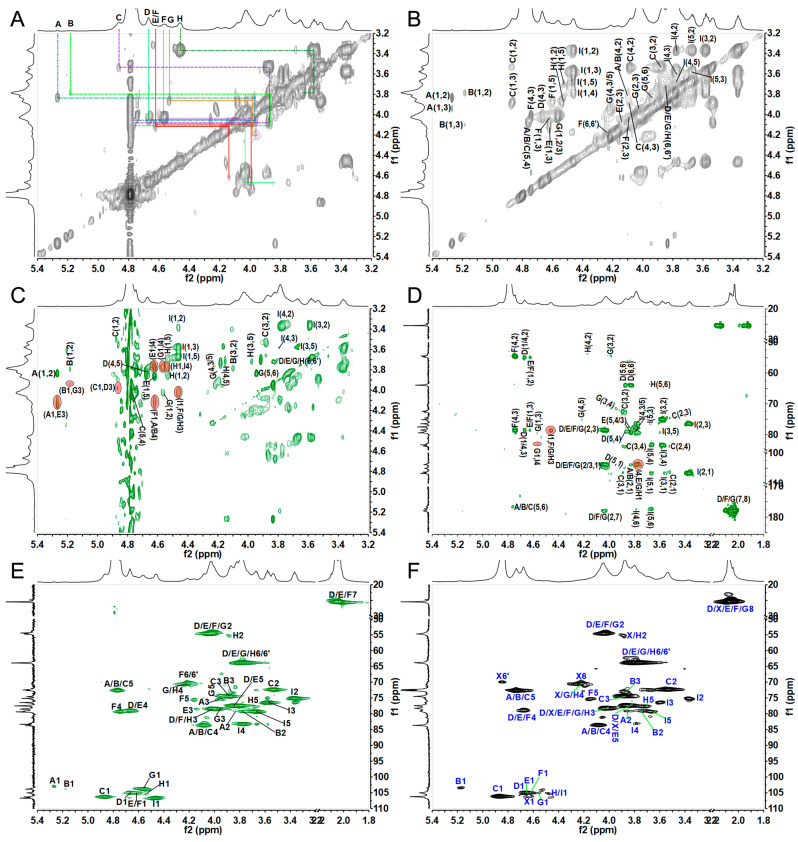
The COSY (**A**), TOCSY (**B**), ROESY (**C**), ^1^H–^13^C HMBC (**D**), and HSQC (**E**) spectra of CMG-1.0 (**A**), and HSQC (**F**) spectrum of GMG-1.0. The correlation signals in red ellipses in (**C**,**D**) indicate the connection positions between sugar residues.

The detailed structural features of CMG-1.0 and GMG-1.0 were further elucidated by 1D/2D NMR analyses. First, some structural information obtained from the above physicochemical analyses can be further confirmed by the ^1^H and ^13^C NMR spectra (Figure 2 and Figure 3). According to the literature [29], the relatively downfield chemical shifts (>4.8 ppm) of the anomeric signals suggested the α configuration of residues A–C. Relatively upfield chemical shifts (<4.7 ppm) of the anomeric proton signals indicated β configuration of residues D–I. The signals (5.27, 5.18, and 4.86 ppm) in the anomeric region may be from α-L-IdoA residues, while the anomeric signal of 4.47 ppm may be due to β-D-GlcA residues, according to the literature [29,30]. In addition, the anomeric protons at 4.67, 4.62, 4.61, 4.56, and 4.53 ppm may be attributed to the β-D-GalNAc residues. The intense signals with upfield resonance appeared at around 2.03–2.07 ppm, which may be due to the acetyl methyl groups in the amino sugars, such as GlcNAc and GalNAc residues in these GAGs. The amino sugar residues D–G were almost acetylated according to their peak area integration of the anomeric proton and the methyl from the acetyl groups. In the ^13^C spectra, the most downfield resonance, δ_H_ at 176–178 ppm, can be ascribed to two carbonyl groups in IdoA, GlcA, GlcNAc, and GalNAc residues. The anomeric carbon signals were at 103–107 ppm. The relative upfield signals appearing at approximately 55 ppm can be arbitrarily assigned as C-2 resonance of GlcNAc and GalNAc residues because of the presence of the amino group at this position. The most upfield signals at approximately 25 ppm may be attributed to the acetyl methyl groups in the GlcNAc and GalNAc residues. Subsequently, the 2D NMR spectra (^1^H–^1^H COSY, TOCSY, ROESY, ^1^H–^13^C HSQC, HSQC-TOCSY, and HMBC) (Figure 4 and Figures S4–S6) were applied to assign all the chemical shifts of various residues compared with the data available in the literature [29,30,31,32]. The assignment results are shown in Table 1.

CMG-1.0 showed nine intra-residue spin coupling systems in ^1^H–^1^H COSY, TOCSY, and ROESY spectra (Figure 4A–C), indicating that it contained nine kinds of sugar residues linked to various sugar residues. The obvious signals at 5.27/103.6, 5.18/103.9, 4.86/106.3, 4.67/105.1, 4.62/105.0, 4.61/105.0, 4.56/103.8, 4.53/105.6, and 4.47/106.7 ppm in the HSQC spectrum were assigned to the anomeric signals of various sugar residues designated as A, B, C, D, E, F, G, H, and I, respectively (Figure 4E). The cross-signals at 4.05/54.3, 4.08/54.7, 4.10/54.3, 4.02/54.5, and 3.86/55.1 ppm in the HSQC spectrum can be readily assigned to H-2/C-2 of the D–H residues, which indicated that they were β-D-GalNAc or β-D-GalN residues. Chemical shifts of H-2 of A–I can be readily obtained from the COSY spectrum, and their C-2 chemical shifts can be assigned by the HSQC spectrum. The residues A, B, C, and I were then identified as the α-L-IdoA or β-D-GlcA residues because they had a relatively large C-2 signal compared with the amino sugar. The proton signals of the nine systems from H-3 to H-6 can also be assigned carefully using the ^1^H–^1^H COSY, TOCSY, and ROESY spectra, although some signals in these spectra are weak. The downfield chemical shift of H-5 of residues A–C (δ_H_ > 4.7 ppm) further confirmed that they were L-IdoA residues for their C-5 epimerization [31]. Then, the residue I was confirmed to be the D-GlcA residue. The detailed carbon signals of various sugar residues from C-3 to C-6 were assigned based on the assignment of the protons using the ^1^H–^13^C HSQC and HSQC-TOCSY spectra (Figure 4E and Figure S4). Therefore, all signals from the 1D/2D NMR spectra can be clearly assigned, as shown in Table 1.

The sequence of sugar residues in CMG-1.0 was confirmed by the ROESY and HMBC spectra (Figure 4C,D). For example, the cross signal (4.86, 4.03 ppm) in the ROESY spectrum showed that residue C had a strong inter-residue ROE connected to H-3 of residue D, confirming that residue C was linked to the C-3 position of residue D. The cross signal (4.67, 4.08 ppm) indicated that residue D was linked to the C-4 position of residue C. Similarly, the linkages (I-β1,3-F/G/H), (E/G/H-β1,4-I), (A-α1,3-E), (B-α1,3-G), (F-β1,4-A/B) were confirmed by the cross peaks at (4.47, 4.00 ppm), (4.62/4.56/4.53, 3.78 ppm), (5.27, 4.14 ppm), (5.18, 3.96 ppm), and (4.61, 4.10/4.13 ppm) in the ROESY spectrum. The β1,3-linkages between residue I and residues F/G/H were further confirmed by the correlation signals (4.47, 78.6 ppm) in the HMBC spectrum. The cross signals (3.78, 103.8 ppm) further confirmed the β1,4-linkages between residue G and residue I.

The down-field chemical shifts of protons and carbons caused by the sulfation can identify the sulfated positions on residues A–I in comparison with the corresponding unsubstituted monosaccharide. Compared with the H/C-4 chemical shifts (4.21/70.3, 4.17/70.3 ppm) of residues G and H, the downfield H/C-4 chemical shifts (4.66/79.2, 4.63/79.1, 4.74/79.4 ppm) of residues D, E, and F indicated that these positions were sulfated. Furthermore, the downfield chemical shift of H/C-6 (4.17/4.25, 70.0 ppm) confirmed that residue F was sulfated at the C-6 position. Similarly, the downfield H/C-2 chemical shifts (3.84/79.1, 3.79/79.2 ppm) of residues A and B were obviously higher than the corresponding chemical shift (3.53/72.4 ppm) of residue C, indicating that C-2 positions of residues A and B were sulfated.

**Figure 5:**
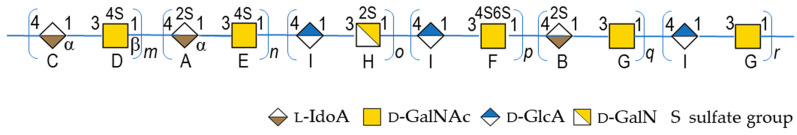
The proposed polysaccharide sequence of CMG-1.0 and GMG-1.0.

Based on the above analysis, the proposed polysaccharide sequence of CMG-1.0 was -{-C-α1,3-D-β1,4-}_m_-{-A-α1,3-E-β1,4-}_n_-{-I-β1,3-H-β1,4-}_o_-{-I-β1,3-F-β1,4-}_p_-{-B-α1,3-G-β1,4-}_q_-{-I-β1,3-G-β1,4-}_r_ (Figure 5). According to the integral area of the anomeric proton of amino sugars in the ^1^H spectrum (Figure 2A), the proportion of various disaccharide units in the CMG-1.0 can be calculated to be m:n:o:p:q:r = 26:6:2:2:1:9.

Based on the above results of the NMR analysis of CMG-1.0, the detailed chemical structure of GMG-1.0 was also further confirmed by its 1D/2D NMR analysis (Figure 4F, Figures S5 and S6). The ^1^H and ^13^C NMR spectra and the chemical shifts from identified 2D NMR spectra are shown in Figure 2B and Figure 3B and Table S1. It was observed that most signals from the same residues of GMG-1.0 were similar to those of CMG-1.0. GMP-1.0 was proposed to have a similar polysaccharide sequence to CMG-1.0, except that the GMP-1.0 obviously had a different disaccharide proportion of m:n:o:p:q:r = 69.5:6:3:3.5:1:1. These results indicated that both CMG-1.0 and GMG-1.0 were rich in DS domain, i.e., they were mainly composed of repeating disaccharide units of -{IdoA-α1,3-GalNAc_4S_-β1,4-}-. However, the proportions of various disaccharide units in these two GAGs were obviously different, which may be due to the different species. CMG-1.0 had a higher proportion of CS-O disaccharide unit -{-GlcA-β1,3-GalNAc-β1,4-}- but a lower proportion of CS-E disaccharide unit -{-GlcA-β1,3-GalNAc_4S6S_-β1,4-}- than GMG-1.0.

Based on the results of monosaccharide, disaccharide composition, and NMR analyses, CMG-1.0 and GMG-1.0 were confirmed to be CS/DS hybrid chains with high amounts of DS-A disaccharide units. Since some disaccharide units are extremely low in these CS/DS chains, especially in the GMG-1.0, with a low resolution of the ROESY and HMBC spectra, the precise structure of these GAGs may be elucidated by analyzing their oligosaccharide fragments, as carried out in our previous studies [10,33].

DS often occurs in co-polymeric form with CS, thus forming a CS/DS hybrid chain. To date, many CS/DS hybrid chains with varying proportions of CS and DS disaccharide units have been isolated from marine animals, such as shark skin and brittlestars [33,34]. These hybrid chains from different species of marine animals displayed enormous structural diversity mainly due to the variability of sulfate substitution and showed multiple biological activities, such as neuritogenic activity, wound healing, and anticoagulant activity, which had potential as therapeutic agents [34,35,36]. In 2017, GAGs from fish swim bladders were determined to contain 95% CS of the total GAG [22]. Considering that the CS may contain a CS-B (DS) unit, it was probably a CS/DS hybrid chain. The integration of the peak area for 1H–IdoA and 1H–GlcA in the ^1^H NMR spectrum suggested that the ratio of DS disaccharide unit to CS-A disaccharide unit was 1:1.4. In our present studies, the CS/DS hybrid chains CMG-1.0 and GMG-1.0 are mainly composed of DS-A disaccharide unit and a small amount of DS-B and CS disaccharide units. Of note, a very small amount of β-D-GalN_2S_ residue was also found in CMG-1.0 and GMG-1.0, which is rare in natural CS/DS. Therefore, the structures of CMG-1.0 and GMG-1.0 are obviously different from those of the CS/DS hybrid chains in previous reports [20,22].

### 2.8. Anticoagulant Activity

The anticoagulant activities of CMG-1.0 and GMG-1.0 were investigated by the classical coagulation assays, such as the activated partial thromboplastin time (APTT), thrombin time (TT), and prothrombin time (PT) assays, and the results are shown in Table 2. Both CMG-1.0 and GMG-1.0 showed significant anticoagulant activity by prolonging APTT. The concentration of 0.226 μM of GMG-1.0 was required to double the APTT, indicating that it had a strong intrinsic anticoagulant activity that was stronger than that of low-molecular-weight heparin (LMWH). The concentration of GMG-1.0 required to double the TT was 12.035 μM, indicating that it can obviously inhibit the common pathway of the coagulation cascade. CMG-1.0 did not exhibit TT and PT prolonging activity, indicating it had a higher selective to inhibit the intrinsic coagulation pathway than GMG-1.0. Both CMG-1.0 and GMG-1.0 showed no PT prolonging activity, indicating that they had no effect on the extrinsic coagulation pathways. The obviously different anticoagulant activity of these two GAGs may be due to their different structural units and proportions.

Considering that CMG-1.0 and GMG-1.0 can obviously prolong APTT, they may have the potential to inhibit the coagulation factors, such as factor XIIa, factor XIa, factor IXa, and intrinsic tenase associated with the intrinsic coagulation pathway. The activity assay revealed that GMG-1.0 potently inhibited the intrinsic tenase with an EC_50_ value of 58 nM (Table 2). The intrinsic tenase is the rate-limiting enzyme in the intrinsic pathway, and inhibitors of this enzyme complex, such as depolymerized products and nonasaccharide from fucosylated glycosaminoglycans, exhibit strong anticoagulant and antithrombotic activities while avoiding adverse effects [33,37]. The reason is that intrinsic tenase inhibition has no effect on the extrinsic coagulation pathway and preserves the hemostatic function [38]. Therefore, the intrinsic tenase has been recognized as a potential target for developing anticoagulant inhibitors. The application value of GMG-1.0 as a potent and safe intrinsic tenase inhibitor to prevent thrombus formation deserves further investigation.

## 3. Materials and Methods

### 3.1. Materials and Reagents

Two commercial dried swim bladders were purchased from a local seafood market in Nanning city and identified as *C. microlepidotus* and *G. morhua* by Professor Jing Wen at Shaoguan University. The species identification results are shown in the Supplementary File. D-GlcA, D-GalA, D-glucose (Glc), D-Gal, D-GalNAc, D-GlcNAc, and Chondroitinase ABC from Proteus vulgaris (EC 4.2.2.4) were obtained from Sigma–Aldrich (St. Louis, MO, USA). L-IdoA was purchased from Shanghai ZZBIO Co., Ltd. (Shanghai, China). Alcian blue 8GX, D-mannose (Man), and L-fucose (Fuc) were obtained from Aladdin Chemical Reagent Co., Ltd. (Shanghai, China). Standard pullulans were obtained from Sepax Technologies, Inc. (Delaware, USA). Deuterium oxide (D_2_O) with 99.9% atom D and D_2_O containing 0.05 wt% 3-(trimethylsilyl) propionic-2,2,3,3-d4 acid (TSP) sodium salt were obtained from Sigma-Aldrich (Beijing, China). CS and DS disaccharide standard mix (CD Mix), including ΔDi0S (ΔUA-1,3-GalNAc), ΔDi6S (ΔUA-1,3-GalNAc_6S_), ΔDi4S (ΔUA-1,3-GalNAc_4S_), ΔDi2S (ΔUA_2S_-1,3-GalNAc), ΔDi2,6diS (ΔUA_2S_-1,3-GalNAc_6S_), ΔDi4,6diS (ΔUA-1,3-GalNAc_4S6S_), ΔDi2,4diS (ΔUA_2S_-1,3-GalNAc_4S_), and ΔDi2,4,6triS (ΔUA_2S_-1,3-GalNAc_4S6S_) were obtained from Iduron (Manchester, UK). Enoxaparin (M_w_: 4500 Da, 0.4 mL × 4000 AXaIU) was purchased from Sanofi-Aventis (Paris, France). Tris-HCl (>99.5%) was purchased from Amresco (USA). Coagulation control plasma, 0.05 M CaCl_2_ solution, and APTT, PT, and TT assay kits were obtained from TICO GmbH (Hamburg, Germany). Human FVIII was obtained from Shanghai RAAS Blood Products Co., Ltd. (Shanghai, China). Biophen FVIII: C kit was from Hyphen Biomed (Paris, France). All other chemicals and reagents used were of analytical grade.

### 3.2. Extraction and Isolation of GAGs from Swim Bladder

The dried swim bladders were ground to powder using a homogenizer. GAGs were extracted according to a procedure described by Vieira et al. with slight modifications [39]. Briefly, the swim bladder powder was suspended in distilled water (1 g/10 mL) and treated with 1% papain solution at 55 °C for 16 h. The mixture was digested by NaOH solution at the final concentration of 0.5 M at 60 °C for 2 h. After cooling to room temperature, the pH of the mixture was adjusted to 2–3 by the addition of 6 M HCl solution. The supernatant was obtained by centrifugation, and the pH was adjusted to 7.0. Ethanol was then added to the final concentration of 75% (*v*/*v*), standing overnight at 4 °C. Finally, the precipitate was collected after centrifugation at 4816× *g* for 15 min and lyophilization.

The crude GAGs were dissolved in distilled water, applied to a column packed with Amberlite FPA98Cl anion-exchange resin, and eluted with increasing concentrations of NaCl solution (0, 0.5, 1.0, 1.5, 2.0 M). The main acidic fractions eluted by 1.0 M NaCl were collected, precipitated by ethanol, desalted by a dialysis bag with molecular weight cut-off of 3.5 kDa, and lyophilized to obtain white powders named CMG-1.0 and GMG-1.0.

### 3.3. Physicochemical Analysis

Protein contents in the CMG-1.0 and GMG-1.0 samples were determined by the method described by Bradford [40] using bovine serum albumin as a standard. The uronic acid content was determined using the Blumenkrantz and Asboe-Hansen procedure [41] using GalA as a standard. The sulfate group content was measured by the turbidimetric method [42]. The sulfate/carboxyl ratio was determined by a conductimetric method, as described in our previous study [43].

The purity and *M*_w_ of GAG were estimated by HPGPC as described in our previous study [26]. The samples were injected into the Shodex OHpak SB-804 HQ column (7 µm, 8 × 300 mm) and eluted with 0.1 M NaCl at a flow rate of 0.5 mL/min. For the *M*_w_ calculation, a standard curve was made using standard pullulans with *M*_w_ of 1.08, 5.9, 9.6, 21.1, 47.1, 107, and 200 kDa.

Cellulose acetate electrophoresis was performed as reported previously [36,44] with minor modifications. Briefly, the samples and standard GAGs, such as HP, CS, and DS, were prepared at a concentration of 5 mg/mL. The cellulose acetate strips were stated in 50% methanol overnight and then soaked in electrophoretic buffer (0.1 M barium acetate buffer, pH 5.0) for 30 min. The samples and GAG standards were placed at the origin of the cellulose acetate strip and ran in the electrophoretic buffer for 1 h 55 min. After migration, the strip was stained with alcian blue for 15 min, and the excess stain was then removed by soaking in 2% acetate buffer for 10 min.

PMP pre-column derivatization combined with HPLC was used to analyze the monosaccharide composition [45]. Briefly, samples were hydrolyzed with 4 M TFA. The released monosaccharides were then derivatized by PMP and further analyzed by HPLC equipped with an Agilent ZORBAX Eclipse Plus C18 column (4.6 × 250 mm, 5 μm). The unknown peak in the HPLC profiles of derivatives was further analyzed by ESI-Q-TOF-MS. The identification was performed on an ACQUITY UPLC BEH C18 column (2.1 × 100 mm; 1.7 μm) using an ACQUITY UPLC I-Class and Xevo G2-XS QTOF HRMS spectrometer (Waters, USA). The analytical conditions of the MS were as follows: ESI in a positive-ion mode, capillary voltage of 3.0 kV, source temperature of 115 °C, source offset of 80, desolvation temperature of 450 °C, cone gas flow of 50.0 L/Hr, desolvation gas flow 800.0 L/Hr, and MSMS collision energy of 15–35 eV. The mass spectrum was acquired in scan mode (*m*/*z* scan range 100–1500).

FT-IR spectra were determined by the methods described previously [46]. The spectra were scanned from 4000 to 400 cm^−1^ with an iS50 infrared spectrometer (Thermo Fisher Scientific, Waltham, MA, USA).

### 3.4. Enzymatic Treatment and Disaccharide Composition Analysis

The disaccharide composition analysis was carried out as previously reported [47]. Briefly, the sample was incubated with chondroitin ABC lyase at 37 °C for 48 h. After heating in boiled water for 5 min, the mixture was centrifugated to obtain the supernatant and analyzed by the SAX-HPLC (Welch Ultimate XB-SAX, 4.6 × 250 mm). The mobile phase was a mixture of 2 mM Na_2_HPO_4_ (pH 3.0, solvent A) and 2 mM Na_2_HPO_4_ containing 1.2 M NaClO_4_ (pH 3.0, solvent B). The gradient was programmed as 97% A at the beginning, and the mobile phase B linearly increased from 3% to 35% during 120 min. The flow rate was 0.6 mL/min, and the detection wavelength was 232 nm.

### 3.5. NMR Spectroscopy

The dried samples (10–20 mg) were dissolved in 0.5 mL of D_2_O without the internal standard TSP and freeze-dried thrice to replace the exchangeable protons with deuterium. The samples were then redissolved in 0.5 mL of D_2_O containing TSP for NMR analysis. The NMR spectra were recorded on a Bruker AVANCE NEO 600 M spectrometer at 298 K. The ^13^C spectra were recorded with a number of scans of 16,384. The 2D NMR data were collected using an 11 ppm spectral width, 1024 data points in the direct dimension, and 160 increments in the indirect dimension with 2–24 scans. A relaxation delay of 1.5 s was used. All chemical shifts were relative to the internal TSP (δ_H_ and δ_C_ = 0.00).

### 3.6. Assay of Anticoagulant Activity

The anticoagulant activity of the CMG-1.0 and GMG-1.0 samples was investigated by the APTT, TT, and PT assays as described in our previous study [46]. The APTT assay was carried out by mixing 5 μL of samples at various concentrations and 45 μL of standard human plasma and incubating at 37 °C for 2 min. Fifty microliters of APTT reagent were then added, and the mixture was kept at 37 °C for 3 min. The clotting time was immediately recorded after the addition of 50 μL of 0.02 M CaCl_2_ solution. For PT assay, 5 μL of samples at various concentrations were mixed with 45 μL of standard human plasma at 37 °C and incubated for 2 min. The clotting time was obtained after adding the PT reagent (100 μL). The TT assay was carried out by mixing 10 μL of samples at various concentrations and 90 μL of standard human plasma and incubating at 37 °C for 2 min. The clotting time was recorded after the addition of 50 μL of TT reagent. Tris-HCl buffer and LMWH were used as the blank and positive control, respectively.

The activity of intrinsic tenase inhibition was determined by a colorimetric method using a 96-well plate kinetic assay as previously described [33]. Briefly, equal volumes of sample solution, factor VIII, and factor IXa were mixed and incubated at 37 °C for 2 min. The R1 solution was then added to the mixture and incubated at 37 °C for 1 min. Finally, the R3 solution was added and mixed well, and the absorbance was recorded at 405 nm.

### 3.7. Statistical Analysis

Determinations of chemical composition and biological activity mentioned in Section 3.3 and Section 3.6 were performed in triplicate and twice, respectively. The data were analyzed by the IBM SPSS software (Chicago, IL, USA) and expressed as the mean ± standard deviation (SD). One-way analysis of variance (One-Way ANOVA) and Duncan’s new multiple-range test were used for the statistical analysis. *p* < 0.05 was statistically significant.

## 4. Conclusions

In this study, two crude GAGs were extracted from the swim bladders of *C. microlepidotus* and *G. morhua* by enzymatic and alkaline hydrolysis, and the purified GAGs, CMG-1.0 and GMG-1.0, were obtained after isolation and purification using the anion-exchange chromatography. The detailed structures of these purified GAGs were elucidated by the physicochemical analyses, such as chemical composition, monosaccharide and disaccharide composition analyses, and 1D/2D NMR spectroscopy. The polysaccharide sequences of CMG-1.0 and GMG-1.0 were confirmed to be the CS/DS hybrid chains mainly composed of the repeating disaccharide unit DS-A and a small quantity of DS-B and CS disaccharide units. CMG-1.0 had a higher proportion of CS-O disaccharide units but a lower proportion of CS-E disaccharide units than GMG-1.0. The disaccharide compositions and proportions of GAGs from swim bladders varied in a species-specific manner. Both CMG-1.0 and GMG-1.0 showed potent anticoagulant activity mainly by inhibiting the intrinsic coagulation pathway. Further studies on the anticoagulant mechanism indicated that GMG-1.0 with strong inhibition activity of intrinsic tenase had the potential to be developed as an intrinsic tenase inhibitor. The significant difference in the anticoagulant activity between the CMG-1.0 and GMG-1.0 may be attributed to their differences in their disaccharide compositions and proportions. These findings can provide significant reference for the potential application of swim bladder by-products of marine fish processing in the pharmaceutical industry. To the best of our knowledge, no information is available in the literature on the structural sequence and anticoagulant activity of CS/DS hybrid chains from *C. microlepidotus* and *G. morhua*. More effort is needed to investigate the detailed structure–activity relationships and the action mechanisms of the anticoagulant activity of these CS/DS hybrid chains.

## Figures and Tables

**Table 1:** Assignment of ^1^H and ^13^C signals of CMG-1.0.

Residues	H/C	Chemical Shifts (δ,ppm) ^a^								Connection Patterns Cross Signals (ppm)
		1	2	3	4	5	6	7	8	
A	H	5.27	3.84 ^b^	3.95	**4.10** ^c^	4.76				A-E
α-L-IdoA_2S_	C	103.6	79.1	75.5	**83.0**	72.4	176.8			(5.27, 4.14)
B	H	5.18	3.79	4.10	**4.13**	4.78				B-G
α-L-IdoA_2S_	C	103.9	79.2	75.1	**83.5**	72.4	176.8			(5.18, 3.96)
C	H	4.86	3.53	3.88	**4.08**	4.76				C-D
α-L-IdoA	C	106.3	72.4	74.6	**83.8**	72.7	176.6			(4.86, 4.03)
D	H	4.67	4.05	**4.03**	4.66	3.87	3.73/3.84		2.03	D-C
β-D-GalNAc_4S_	C	105.1	54.3	**78.4**	79.2	77.5	63.9	178.0	25.5	(4.67, 4.08)
E	H	4.62	4.08	**4.14**	4.63	3.83	3.73/3.84		2.05	E-I
β-D-GalNAc_4S_	C	105.0	54.7	**78.6**	79.1	77.7	63.9	177.9	25.5	(4.62, 3.78)
F	H	4.61	4.10	**4.04**	4.74	4.15	4.17/4.25		2.04	F-A/B
β-D-GalNAc_4S6S_	C	105.0	54.3	**78.9**	79.4	75.7	70.0	177.9	25.5	(4.61, 4.10/4.13)
G	H	4.56	4.02	**3.96**	4.21	3.93	3.72/3.84		2.07	G-I
β-D-GalNAc	C	103.8	54.5	**78.6**	70.3	75.7	63.9	177.4	25.5	(4.56, 3.78)
H	H	4.53	3.86	**4.00**	4.17	3.70	3.74/3.82			H-I
β-D-GalN_2S_	C	105.6	55.1	**78.6**	70.3	77.3	63.9			(4.53, 3.78)
I	H	4.47	3.37	3.58	**3.78**	3.67				I-F/G/H
β-D-GlcA	C	106.7	75.2	76.5	**83.3**	79.4	177.3			(4.47, 4.04/3.96/4.00)

^a^ The 600 MHz NMR spectra were recorded at 298 K. All chemical shifts are relative to TSP at 0 ppm. ^b,c^ Values underlined and boldface indicate sulfated and glycosylated positions, respectively.

**Table 2:** Effects of CMG-1.0 and GMG-1.0 on APTT, PT, TT, and intrinsic tenase (*n* = 2).

Sample	*M*_w_ (kDa)	APTT ^1^, μM	TT ^1^, μM	PT ^1^, μM	anti-tenase ^2^, μM
LMWH	~4.5	0.571 ± 0.003	0.658 ± 0.043	/ ^3^	0.010 ± 0.001
CMG-1.0	109.3	0.586 ± 0.001	/	/	/
GMG-1.0	123.1	0.226 ± 0.016	12.035 ± 0.404	/	0.058 ± 0.015

^1^ The activity of samples to prolong APTT, PT, or TT is expressed by each drug concentration (μM) required to double the APTT, PT, or TT; ^2^ EC_50_ value, the concentration of each sample required to inhibit 50% of tenase activity; ^3^ not determined.

## Data Availability

The original data presented in the study are included in the article/Supplementary Material; further inquiries can be directed to the corresponding author.

## Supplementary Materials

The following supporting information can be downloaded at: https://www.mdpi.com/article/10.3390/md22010009/s1, Figure S1: UV spectrum of CMG-1.0 and GMG-1.0; Figure S2: Electrophoretogram of CMG-1.0 (Track 2) and GMG-1.0 (Track 3); Figure S3: FT-IR spectra of CMG-1.0 and GMG-1.0; Figure S4: ^1^H-^13^C HSQC-TOCSY spectrum of CMG-1.0; Figure S5: ^1^H-^1^H COSY spectrum of GMG-1.0; Figure S6: ^1^H-^1^H TOCSY spectrum of GMG-1.0; Table S1: Assignment of ^1^H and ^13^C NMR signals of GMG-1.0; Species identification results.
