# Supplementary material for: Chondroitin Sulfate/Dermatan Sulfate Hybrid Chains from Swim Bladder: Isolation, Structural Analysis, and Anticoagulant Activity
Source: Mar Drugs. 2023 Dec 21;22(1):9. doi: 10.3390/md22010009 (PMC10817686; doi:10.3390/md22010009)
Supplement: Supplementary file 1 [file marinedrugs-22-00009-s001.zip › marinedrugs-2724374-supplementary.pdf]

Supplementary Data

Chondroitin Sulfate/Dermatan Sulfate Hybrid Chains from Swim Bladder: Isolation, Structural Analysis, and Anticoagulant Activity

Yue Yao [1,2,†], Hao Tang [1,†], Haiqiong Ma [1], Zidong Liu [1], Jinwen Huang [1], Xiufen Yang [2,*], Longyan Zhao [1,*] and Qingxia Yuan [1,*]

[1] Guangxi Key Laboratory of Marine Drugs, Institute of Marine Drugs, Guangxi University of Chinese Medicine, Nanning 530200, China
[2] School of Pharmacy, Guangxi University of Chinese Medicine, Nanning 530200, China
* Correspondence: xiufenyang@163.com (X.Y.); longyanzhao@gmail.com (L.Z.); qingxiayuan@163.com (Q.Y.)
† These authors contributed equally to this work.

.

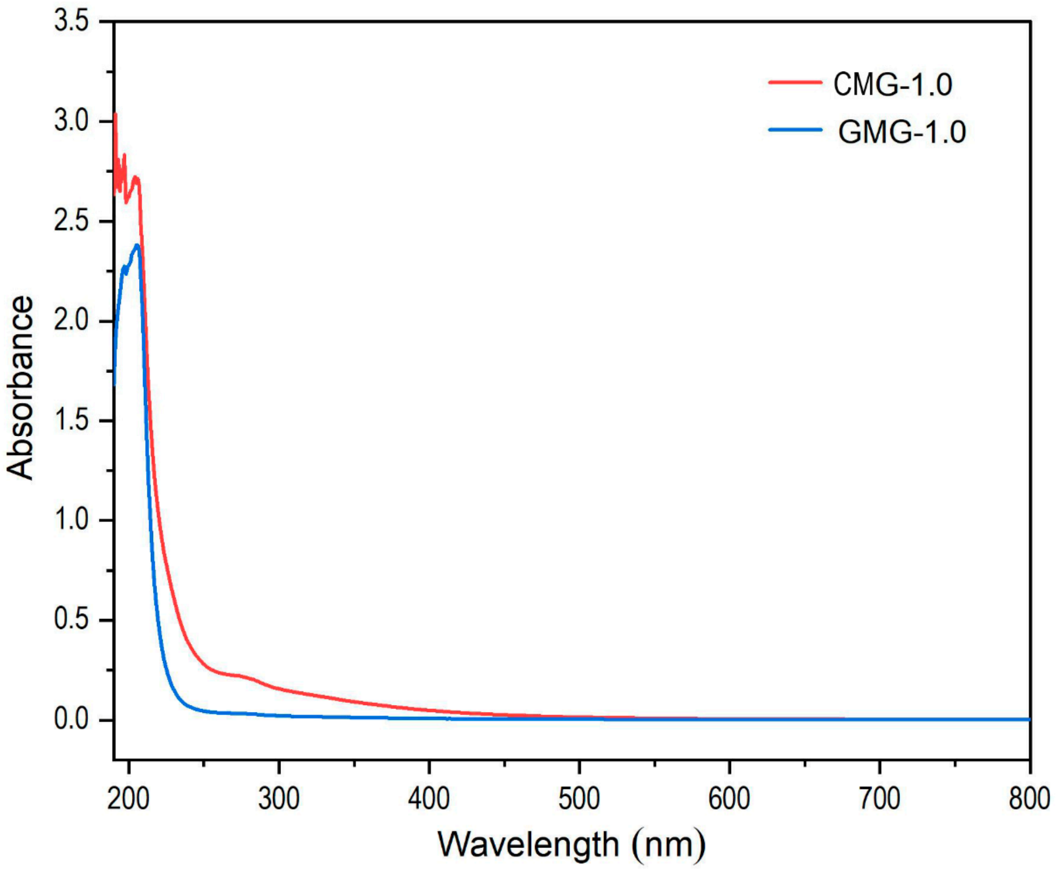

Figure S1. UV spectrum of CMG-1.0 and GMG-1.0

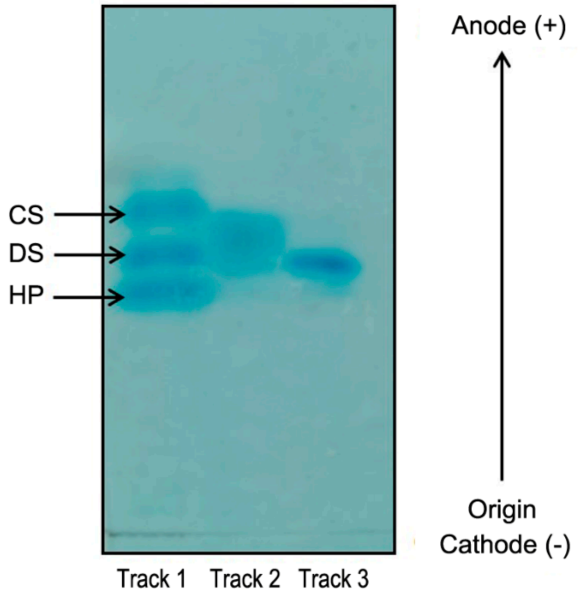

Figure S2. Electrophoretogram of CMG-1.0 (Track 2) and GMG-1.0 (Track 3)

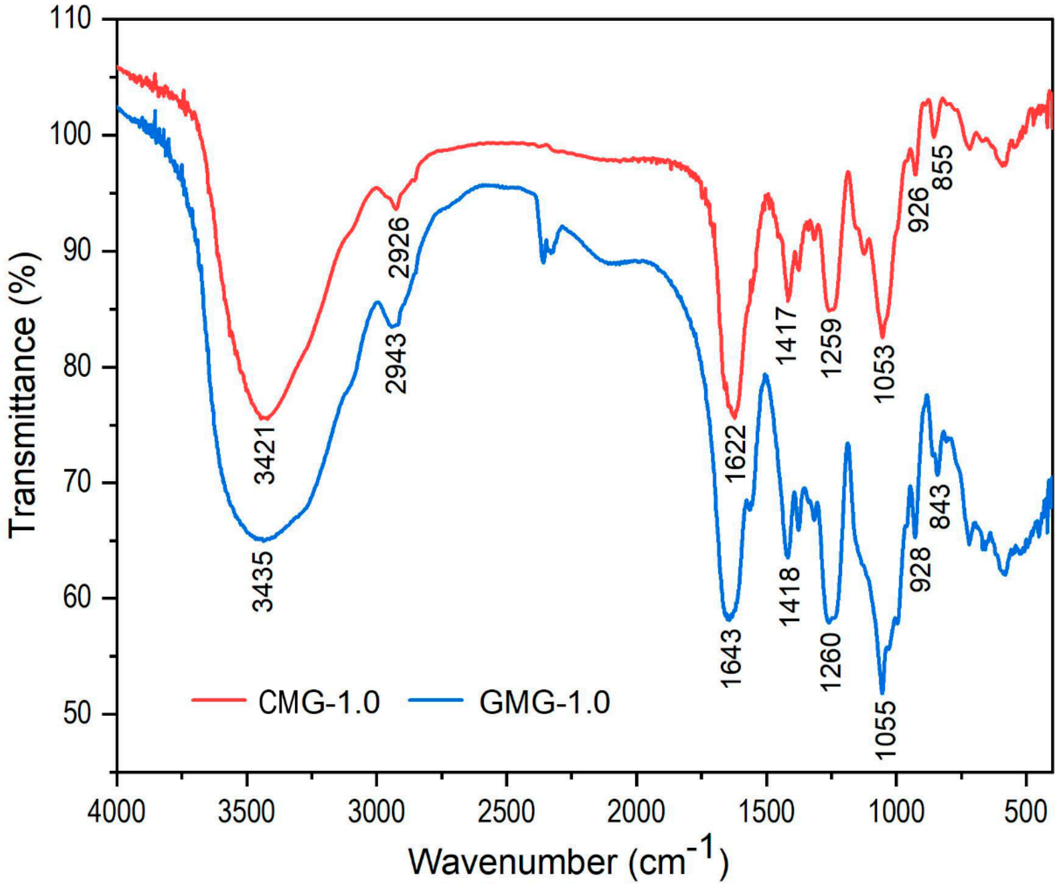

Figure S3. FT-IR spectra of CMG-1.0 and GMG-1.0

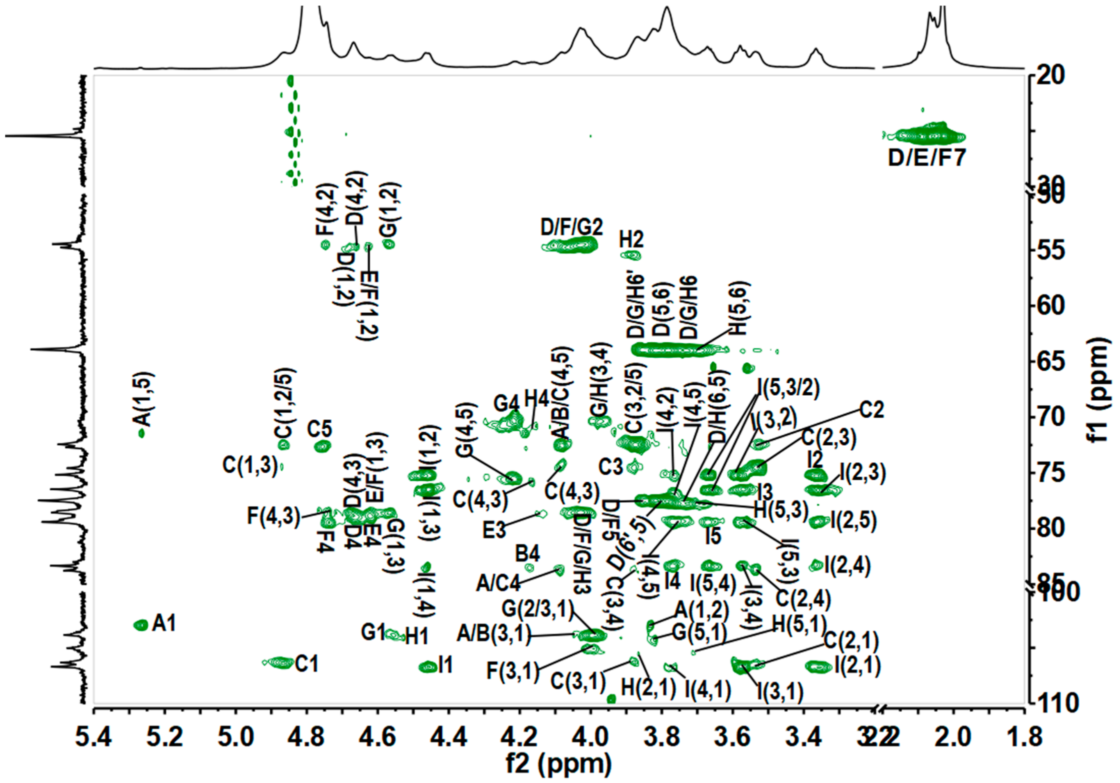

Figure S4. ^{1}H-^{13}C HSQC-TOCSY spectrum of CMG-1.0

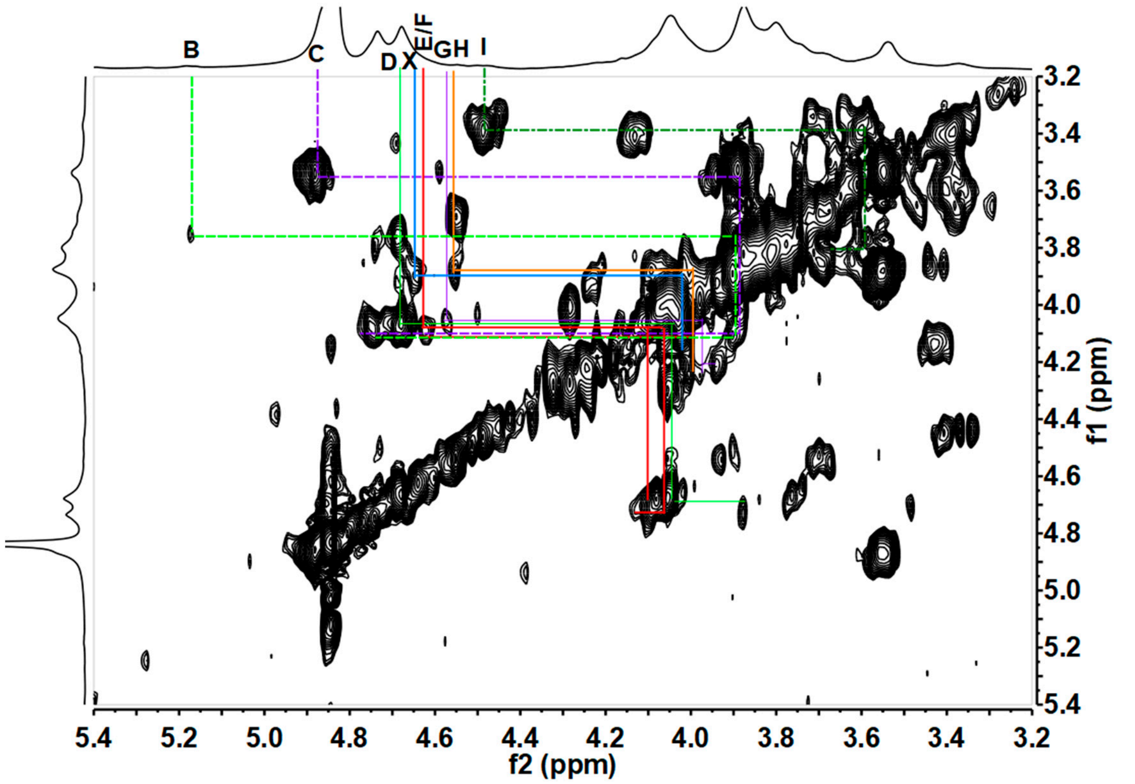

Figure S5. ^{1}H-^{1}H COSY spectrum of GMG-1.0

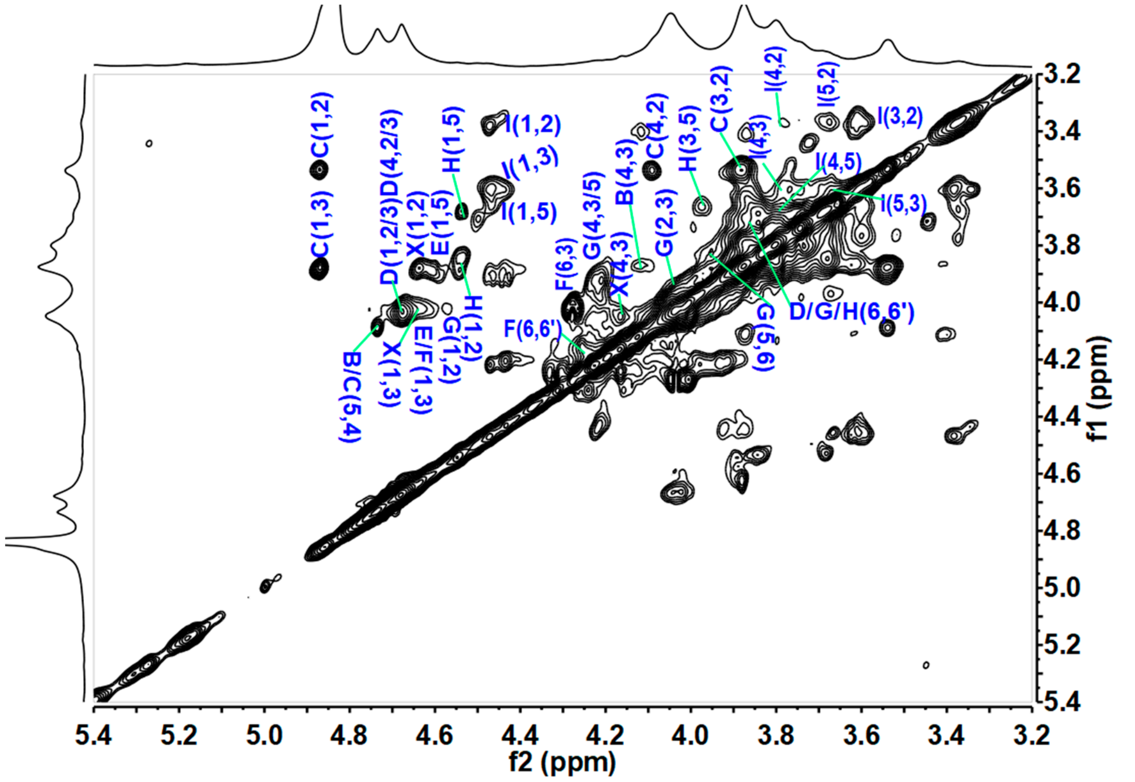

Figure S6. ^{1}H-^{1}H TOCSY spectrum of GMG-1.0

Table S1. Assignment of ^{1}H and ^{13}C NMR signals of GMG-1.0.

Residues	H/C	Chemical Shifts (δ,ppm) [a]							
		1	2	3	4	5	6	7	8
A	H	5.27	3.85 [b]	n.d.	**4.10** [c]	4.76			
α-D-IdoA$_{2S}$	C	103.6	79.0	n.d.	**83.0**	72.4	176.8		
B	H	5.18	3.76	3.87	**4.12**	4.74			
α-D-IdoA$_{2S}$	C	103.2	79.1	73.5	**83.7**	72.7	176.8		
C	H	4.87	3.54	3.89	**4.08**	4.74			
α-D-IdoA	C	106.2	72.4	74.5	**83.7**	72.7	176.8		
D	H	4.67	4.08	**4.04**	4.68	3.87	3.74/3.84		2.08
β-D-GalNAc$_{4S}$	C	105.0	54.3	**78.1**	78.9	77.4	63.8	178.1	25.0
X	H	4.64	3.89	**4.02**	4.16	3.85	4.17/4.84		2.08
β-D-GalN$_{2S6S}$	C	105.3	55.0	**78.9**	70.8	77.4	70.0	178.1	25.0
E	H	4.62	4.10	**4.08**	4.65	3.88	3.73/3.84		2.05
β-D-GalNAc$_{4S}$	C	105.0	54.7	**78.6**	79.1	77.7	63.9	177.9	25.5
F	H	4.61	4.08	**4.04**	4.73	4.15	4.17/4.25		2.04
β-D-GalNAc$_{4S6S}$	C	105.0	54.3	**78.9**	79.4	75.7	70.0	177.9	25.5
G	H	4.56	4.02	**3.96**	4.21	3.93	3.74/3.84		2.04
β-D-GalNAc	C	105.0	54.5	**78.6**	70.3	77.4	63.9	177.4	25.5
H	H	4.54	3.88	**4.00**	4.19	3.69	3.70/3.80		
β-D-GalN$_{2S}$	C	105.4	55.0	**78.4**	70.8	77.4	63.8		
I	H	4.48	3.36	3.59	**3.78**	3.67			
β-D-GlcA	C	106.5	75.1	76.4	**83.3**	79.1	177.4		

n.d. -not detected. [a] The 600 MHz NMR spectra were recorded at 298 K. All chemical shifts are relative to TSP at 0 ppm. [b,c] Values with underline and in boldface indicate sulfated and glycosylated positions, respectively.

Species Identification Results

Cynoscion microlepidotus was confirmed by the partial sequence of mitochondrial 16S rRNA gene sequence:

CCTCTACCTAGTTTTTGGTGCATGAGCCGGAATAGTGGGCACAGCTTTGAGCCTTCTG
ATTCGAGCAGAACTCAGTCAACCAGGCGCACTCCTCGGAGACGACCAGGTTTATAAC
GTAATCGTTACAGCACACGCCTTTGTTATAATTTTCTTTATAGTAATGCCCATCATGAT
CGGAGGGTTTGGAAACTGACTTATCCCGCTAATGATCGGAGCCCCCGACATGGCATT
CCCCCGAATAAATAATATGAGCTTCTGACTTCTACCACCCTCCTTCCTCCTACTCTTAA
CCTCATCAGGGGTAGAAGCAGGGGCCGGAACGGGCTGAACAGTTTATCCCCCACTTG
CGGGCAATCTAGCACATGCAGGGGCTTCCGTCGACTTAGCCATCTTCTCCCTGCATCT
CGCAGGTGTGTCCTCGATTCTAGGGGCTATCAACTTCATTACAACCATTATTAATATG
AAACCTCCCGCTATCTCCCAGTATCAGACACCTCTGTTTGTATGGGCCGTCCTAATTA
CAGCTGTCCTCTTACTGCTCTCGCTCCCTGTCTTAGCTGCCGGTATTACAATACTCCTT

ACAGACCGCAATCTCAACACAACCTTCTTCGACCCCGCAGGAGGGGGGACCCAATT
CTCTATCAACACTTATTC

Gadus morhua was confirmed by the partial sequence of mitochondrial 16S rRNA gene sequence:

CGCCTCTTGCTCCAAAAATATAAGAGGTCCCGCCTGCCCTGTGACTATAAGTTTAACG
GCCGCGGTATTTTAACCGTGCGAAGGTAGCGTAATCACTTGTCTTTTAAATGAAGACC
TGTATGAATGGCATCACGAGGGCTTAGCTGTCTCCCATCTCCAGTCAATGAAATTGAC
CTCCCCGTGCAGAGGCGGGGATAATTACATAAGACGAGAAGACCCTATGGAGCTTTA
GACCTAAAGTAAGTCACGTTTAACATGCTGTGATAACAGTAAAAACTTAGTGATATTT
ACTGAAGTGTCTTTGGTTGGGGCGACCGCGGGGTAAAACACAACCCCCATGTGGACC
GGGGATATTATCCCTAATACTCAGAGCCTCTACTCCAAGTAACAGAAATTCTGACTTT
TCTGATCCGGTATAACCGATCAACGAACCGAGTTACCCTAGGGATAACAGCGCAATC
CCCTCTCAGAGCCCATATCGACGAGGGGGTTTACGACCTCGATGTTGGATCAGGACA
TCCTAATGGTGCAGCCGCTATTAAGGGTTCGTTTGTTCAACGATTAAAGTCCT

Based on the National Center for Biotechnology Information (NCBI) Genbank, the BLAST analysis revealed that the sequence similarity with *Cynoscion microlepidotus* and *Gadus morhua* sequence was 100%.
